# Health and the Built Environment: Exploring Foundations for a New Interdisciplinary Profession

**DOI:** 10.1155/2012/958175

**Published:** 2012-08-21

**Authors:** Jennifer Kent, Susan Thompson

**Affiliations:** City Futures Research Centre, Faculty of the Built Environment, The University of New South Wales, Sydney, NSW 2052, Australia

## Abstract

The supportive role of the built environment for human health is a growing area of interdisciplinary research, evidence-based policy development, and related practice. Nevertheless, despite closely linked origins, the contemporary professions of public health and urban planning largely operate within the neoliberal framework of academic, political, and policy silos. A reinvigorated relationship between the two is fundamental to building and sustaining an effective “healthy built environment profession.” A recent comprehensive review of the burgeoning literature on healthy built environments identified an emergent theme which we have termed “Professional Development.” This literature relates to the development of relationships between health and built environment professionals. It covers case studies illustrating good practice models for policy change, as well as ways professionals can work to translate research into policy. Intertwined with this empirical research is a dialogue on theoretical tensions emerging as health and built environment practitioners and researchers seek to establish mutual understanding and respect. The nature of evidence required to justify policy change, for example, has surfaced as an area of asynchrony between accepted disciplinary protocols. Our paper discusses this important body of research with a view to initiating and supporting the ongoing development of an interdisciplinary profession of healthy planning.

## 1. Introduction

The supportive role of the built environment for human health is a fast growing area of interdisciplinary research, evidence-based policy development, and related practice. Physical inactivity, social isolation, and obesity are three of the major risk factors for many of the chronic diseases facing contemporary society. A recent comprehensive review of the burgeoning literature on healthy built environments [1] identified three key built environment domains that support human health.

- The built environment can support physical activity. Some of the ways that this may occur include integrating land use and public transport to promote walking and cycling for transport; preserving a variety of open spaces for recreational use; designing street networks and providing infrastructure for walking and cycling for both recreation and transport.
- The built environment can connect and strengthen communities. Some of the ways that this may occur include providing streets and public spaces that are safe, clean, and attractive; encouraging residential development that is integrated, yet private; enabling community empowerment through meaningful participation in land use decisions.
- The built environment can provide equitable access to healthy food. Some of the ways that this may occur include reducing fast-food exposure in the vicinity of school environments; retaining periurban agricultural lands as a source of easily accessed healthy food; encouraging the establishment of farmers markets and community gardens.

The evidence on the role of the built environment in protecting and promoting human health is compelling. And yet, despite the strength of this research evidence and closely linked origins, the contemporary professions of public health and urban planning largely operate independently of each other in the neoliberal framework of academic, political, and policy silos [2]. A reinvigorated relationship between professionals in health and the built environment is essential if this research is to be further developed and refined, as well as translated into effective policy and practice. 

Part of this reinvigoration will be to examine and recount the ways public health professionals have already been working with colleagues from the built environment. Case studies illustrating good practice models for policy change, research on motivating and justifying new policy, and methodological and theoretical discourse are the chronicles of a professional revival. In the rush for empirical justifications, these important accounts are easily lost. They are significant, however, in that they provide the basis of a richer understanding of why and how two seemingly disparate professions can work together, continuing to improve their collaborative endeavours. The aim of this paper is to illuminate these accounts to support the ongoing development of the interdisciplinary practice of healthy planning. 

We draw on existing research to do this in three stages. Our paper is prefaced by a novel exploration of the theoretical synchronicity between the traditions of urban planning and health. (In using the term “urban planning” in this paper, we note that there are different descriptors for this discipline. Terminology includes town planning, urban and regional planning, land-use planning, strategic planning, or, simply, planning [3]). This is informed by our interpretation of the emerging methodological and theoretical discourse in the literature. To our knowledge, this reflection represents one of the first attempts to explore any common ground between “theories” of urban planning and health promotion. We then illustrate the emergence of a relationship by examining success stories in utilising empirical research as a catalyst for policy and institutional behavioural change. We have reviewed these stories to suggest some “key ingredients” for reviving and nurturing health and built environment professional relationships. These include dedicated funding, ongoing professional education, and broad interdisciplinary collaboration. Our aim is to support those endeavouring to work collaboratively in creating a built environment that supports the health and well-being of all communities. 

## 2. Methodology

The literature discussed in this paper was identified as part of a larger comprehensive review of literature on the relationship between the built environment and health [1]. The methodology for this review is necessarily transdisciplinary and based on an accepted framework for systematic reviews of research on the built environment and the health of the public [4]. The review was systematic in that it sought to answer a clearly formulated question and employed a systematic method to identify, select, and critically appraise the research. The parameters for this review, as well as the detailed methodology, are explicitly described in [1]. In summary, a search of economic, health, medical, transport, and environmental internet and “grey” literature databases was conducted, and a database of 1,615 references relevant to the built environment and health was subsequently created. These references were then assessed for inclusion in the review and categorised into established key domains of the built environment—physical activity, social interaction, and healthy food access. These domains address three of the major risk factors for contemporary chronic disease—physical inactivity, social isolation, and obesity. Outside of the three key domains initially identified, an additional and emerging theme relating to the translation of research into policy was identified. We labelled this “Professional Development.” The theme encompasses case studies illustrating good practice models for policy change, research on cost benefit analysis, together with market demand to encourage appropriate policy. In addition, there is scholarship on the theoretical underpinnings of healthy built environments. In essence, this theme embodies literature that relates to developing healthy built environment interdisciplinary relationships. An analysis and discussion of this literature is the subject of this paper.

## 3. Building a Theory of ****Healthy Built Environments

### 3.1. The Contemporary Focus of Public Health

There has been a shift in conceptualisations of health and disease from the treatment of illness in the individual to disease prevention and health promotion in populations. This has included increased focus on the impact of environments on collective well-being [5, 6] and on the interdependence of environments and individual behaviour [5, 7–10].

Built environments have subsequently emerged as a focus in health research. This “reinvigoration” of the health-built environment interdisciplinary relationship has been expressed in various themes, from the built environment's impact on opportunities for utilitarian and recreational physical activity [11–15], healthy food access, [16–18] exposure to nature and green space [19, 20], community building [21, 22], as well as noise abatement [23], air pollution [24], and crime [25]. 

Theoretically, this shift reflects the increasingly ecological orientation of the health promotion field [5, 6, 26, 27]. Ecological models of health promotion are underpinned by the understanding that health promoting and preventing interventions need to be considered across multiple levels and contexts. Often these contexts are simplified in the literature as the individual, social, and environment; however more comprehensive theorisations of health ecology also recognise the role of the large-scale economic and political influences that shape local context [28, 29].

The ecological orientation emphasises that the most effective health interventions will be tailored to place [30] and the people living in that place. Interventions will respect that individuals of different ages [31, 32], socioeconomic and cultural backgrounds [33–35], and genders [36, 37] will respond to interventions differently. Furthermore, ecological theories recognise the role of educational programs, policy change, and economic incentives [38, 39] while acknowledging that environmental change can also be a relatively low-cost platform on which to build later targeted interventions [40]. Ecological models are based on the idea that comprehensive approaches to health promotion need to consider interventions at multiple scales and in different contexts [41]. We are most interested in environmental influences on health, as a theoretical space playing host to the reinvigoration of the interdisciplinary relationship between health and built environment professionals. We now turn to a consideration of how urban planning's theoretical context interfaces with health as part of our search for an understanding of how the two disciplines might better work together.

### 3.2. Refocusing Contemporary Urban Planning

Urban planning is often criticised for lacking its own discrete theoretical grounding [42]. As a practical and busy discipline, it operates in highly politicised arenas at numerous levels. Nevertheless, planning is able to rally competing stakeholder demands and opinions, which is a great strength of the discipline. In this professional environment, planning practitioners have learned to adapt and perform, rather than to reflect and question. As a result, the discipline has traditionally borrowed its theories from other specialisations in the social sciences to explore “how” land management decisions are made and “how” these decisions might be translated to spatial and social outcomes [43, 44]. The question of “why” we bother to plan at all, however, has been left relatively underexplored.

In her more recent theoretical explorations, eminent urban and regional planning theorist Patsy Healey revises the components of what she calls “the planning project” [45]. Healey proposes that the motivation to pursue governance with an urban planning orientation is linked to an intrinsically anthropocentric belief that it is worth striving to improve “the human condition" [45, page 18]. The role for urban planning is defined by recognition that “human flourishing depends on giving attention to multiple dimensions of human existence, as realised in particular places” [45, page 17]. Urban planners, therefore, provide the expertise to draw together these dimensions as they exist in place with an ultimate motivation to improve the human condition and promote human flourishing.

Theoretically, acknowledging that we plan to promote human flourishing is to acknowledge that we plan for human health. While this has historically been a central concern of urban planning [44], its explicit recognition has generally been buried deep within the day-to-day milieu of competing agendas. This is somewhat ironic given that much urban planning work has a health objective, such as the management of community exposure to harmful uses, the equitable provision of safe places to live and work, the creation of opportunities to connect to each other and the ability to be mobile—physically and socially. In addition, urban planning's long time focus on environmental sustainability has important planetary health objectives, increasingly recognised as beneficial for human health in the medical literature (see, e.g., The Lancet [46]). And while urban planning has not entirely removed these agendas from the promotion of human flourishing, it has demoted human health to an invisible and unidentified pursuit, thereby diminishing its importance. Accordingly, we propose that in order for the discipline of urban planning to promote health, it must explicitly recognise improving and sustaining human health as a primary objective. 

The spatial and social effects and processes of what is generally considered “good urban planning” are also those advocated by the emerging approach to “healthy planning.” Research exploring the professional urban planner's response to healthy planning guidelines has concluded that healthy planning encompasses the already “accepted wisdom” of the urban planning profession [47, page 102]. Neighbourhoods nested within a walkable catchment of shops and services, connected by safe and efficient public and active transport networks, well serviced with open space and other infrastructure such as footpaths and recreational facilities, have been the intentions of strategic urban planners around the world for at least the last 20 years. A health focus further legitimises the principles and policies urban planners recognise as good professional practice. A more explicit recognition of human health in urban planning theory and practice can therefore be a powerful driver to take the urban planning agenda forward.

Despite these theoretical and practical synchronicities and the mutual benefits of alliance, in reality, we are still struggling to define what a healthy built environment might look like and how health and built environment professionals can work together successfully to create such an environment. The urban planners drafting a regional structure plan, for example, still rarely work in concert with public health officials to explore ways that the region can better support physical activity or access to healthy foods. Explicit legislated mechanisms to include health impacts in the assessment of development proposals are still rare outside of the USA. Case studies of the ways this might be happening around the world, and discourse on the ways disciplinary differences can be transcended, are important in overcoming this struggle and provide an evidence base on which to build. The following section of our paper reviews case studies and discourses in the literature to provide an evidence base to inspire continued exchange to build a healthy planning disciplinary profession.

## 4. From Theory to Practice

We start by reviewing the literature for practical guidance from case study examples and discourse on ways the healthy built environment agenda is being initiated in the professional arena. A defined role for health, the practical and psychological benefits of funding for healthy built environment projects, the role of regulation, and ways of drawing in other stakeholders and agendas are discussed. We then turn to unpick some of the more complex elements of the health-built environment interdisciplinary relationship. For example, when is the evidence of relationships between the built environment and health “good enough” to initiate policy change? How can this evidence be presented to lobby for policy change? 

### 4.1. The Key Ingredients for Healthy Built Environment ****Collaborations

#### 4.1.1. Support Professionals through Education

Wooten advise that healthy built environment interdisciplinary relationships should start with educating professionals [48]. Their recommendation is drawn from implementation outcomes of various health-related urban planning policies in California, USA. Education provides knowledge and skills and creates opportunities for professional rapport. Other studies explore ways that this education process can progress. Botchwey et al., for example, evaluate six predominantly graduate-level courses in the US that address the built environment-health relationship [49]. They describe in detail the key ingredients for a model interdisciplinary curriculum for locally delivered courses designed to educate planners and public health officials. Thompson and Capon provide an Australian assessment of the effectiveness of tertiary healthy built environment education for both urban planners and health students [50]. Pilkington et al. detail a UK-based professional education program based on action learning [51]. The program emphasises not only the practical components of each discipline but also seeks to promote an understanding of the ethics, philosophy, and core values of each profession. 

The role of professional development should be to promote an environment of shared understanding. Using case studies of two active living interventions in Oregon, USA, Dobson and Gilroy warn of the limits to professional development [52]. Health and built environment professionals need not become technical experts in new fields but must work together to capitalise on each other's particular skill sets. This requires understanding, and the development of this understanding should be the focus of professional development rather than the explicit development of a technical skill set. An example of this approach is our ongoing capacity building to support the education of health professionals in New South Wales in Australia [53]. 

#### 4.1.2. Allocate Funding

Budgetary support is a mechanism to implement policy to underpin practice (rather than drawing from existing resources), as well as a way to legitimise health as an urban planning issue. It is an indication of institutional support. In their report on the results of an online survey of health officials in California, USA, Schwarte et al. emphasise the importance of budgetary support simply because it dissolves resentment that may arise from the healthy built environment agenda being an added responsibility for planners and health professionals to consider [54]. In evaluating healthy built environment programs in Melbourne, Australia, Thomas et al. found a key element of the success of programs was employment of a dedicated project officer with skills in engaging management and developing cross-disciplinary alliances [55]. Also in Australia, money from the public health sector is being used to advance healthy planning. This is the case with the Healthy Built Environments Program. Situated in a built environment university faculty, the program receives its core funding from the New South Wales State Government's Health Department [53]. In a New-Zealand-based study, Bullen and Lyne advocate that funding of healthy built environment policy is particularly important in deprived neighbourhoods [56]. This avoids exacerbating existing inequalities. 

Research also suggests, however, that financially supported staff will still require the aide of political will, which is often garnered through community support. In their review of a number of healthy built environment interventions in Nebraska, USA, Huberty et al. recognise the importance of welcoming and actively including volunteers, not least because they indicate the interest of the electorate [57]. Volunteers can complement the work of dedicated staff and also provide the grounded and contextual knowledge so essential to healthy built environments.

#### 4.1.3. Define a Role for Health

A commonly identified struggle in the case study literature is establishing an initial, tangible role for public health professionals in the planning agenda. Wooten et al. suggest that a way forward is for health professionals to provide planners with basic data and analyses to help identify a geography of a community's most critical health concerns [48]. Chen and Florax, for example, use health data to map the impact of increased access to healthy food options on the body mass index of populations across disadvantaged neighbourhoods in Indiana [58]. Their simulations have been used to initiate zoning policies that provide incentives for chain grocers to open in disadvantaged areas. In Florida, USA, McCreedy and Leslie describe the way a health-built environment professional rapport can be initiated through the provision of preliminary assessment data [59]. Allender et al. take this recommendation further, advocating that health statistics backed by cost benefit data are more likely to result in policy change [60]. Another role identified for health professionals includes engaging the media and rallying political commitment [61]. As expressed by a London transport planner discussing sustainable transport: “Health is one of the biggest drivers there is alongside climate change to actually take this agenda forward" [60, page 110]. As previously discussed, the argument for health adds weight to the “good urban planning” agenda. There is evidence that media exposure and the support of senior legislators can be particularly influential in the passage of healthy built environment policy and legislation [62–64].

#### 4.1.4. Utilise Regulation

The need for policy change to be mandated through regulation and law is a recurring theme in case study literature [65]. Often, the implementation of healthy built environment initiatives is dependent on the goodwill and enthusiasm of stakeholders. It is undeniable, however, that the omission of health as a key consideration in land use regulation is a major obstacle to successful healthy built environment interventions [66, 67]. Political and market incentives are frequently bounded by regulatory outcomes, and, as a result, urban planning for health remains an expensive and politically unattractive competing consideration. The significance of this oversight is best summed up by an Australian planner who remarked “From where I sit if it's not in the [State] Planning and Environment Act it does not have to happen" [60]. Currently this legislation is undergoing a comprehensive review [68]. Stakeholders with an interest in advancing healthy planning have made submissions arguing for the inclusion of health and well-being as a principle objective of the revised planning act.

Case study examples of the rare attempts to regulate healthy built environment interventions are therefore important records of what can occur when health is conferred regulatory force through built environment legislation. Legislators in the USA have been particularly proactive in pioneering the development and implementation of regulatory instruments to mandate healthy built environment interventions. Kelder et al.'s discussion of the development and implementation of Texas Senate Bill 19 to mandate physical activity in the State's elementary schools is a good example [69]. A similar regulatory instrument was introduced in neighbouring Arkansas, and this instrument has also been the subject of case study research [70]. Although not tied distinctly to health outcomes, Catlin's commentary on Smart Growth legislation introduced to various jurisdictions across the USA also presents a comprehensive argument for the use of regulation as a catalyst for healthy built environment outcomes [67]. Smart Growth has dominated urban planning agendas in the USA for over a decade, advocating compact, mixed use development, where decreased distances lead to decreased reliance on the private car for transport. Smart growth principles are similar to, but not a mirror image of, healthy built environments (e.g., healthy built environments are not necessarily compact environments). The agenda has been legislated across the USA, and in general it has resulted in amendments to land use patterns. Catlin concludes his commentary with a call for even greater recognition of health as an aim of Smart Growth statutes [67]. 

#### 4.1.5. Draw in Other Stakeholders and Agendas

As collaboration ensues, the contested nature of places and the qualities of people who live, work, and travel within them will become apparent. There will never be a single set of “rules” for managing health outcomes in the built environment. The most achievable and acceptable healthy built environment may not be the most economically productive, the most politically expedient, or even the most environmentally friendly. Akin to the challenging nature of interdisciplinary collaboration, the demands and desires of competing stakeholders will have to be managed through negotiation, willingness to explore new solutions, and, ultimately, an acceptance of compromise [1].

The healthy built environment agenda needs to operate within, rather than alongside existing land use governance structures (governance here is narrowly defined as the exercise of administrative authority). This implies connecting with the processes and regulations that are the domain of traditional town planning, as well as with a multitude of other stakeholders [48]. This action of connection not only garners support for healthy built environments but also it can have the added benefit of connecting health with other high profile agendas, such as climate change [46, 71, 72].

There is a body of literature that explores different stakeholder perspectives of healthy built environments. These include urban planning professionals and local government staff [47, 55]; health and built environment professionals from the public and private sectors [73] retailers [74]; school boards [75]; environmental health officers [54]; legislators [64]; economists [75, 76]; developers [77]; families [78]; youth [59]; engineers [79]; community advocates [57, 61, 65]. The general and, perhaps unsurprising, conclusion from this work—is that stakeholder perspectives are mixed—sometimes they overlap and sometimes they are in complete opposition. It is clear, however, that the interests of all stakeholders, whether congruent or competing, need to be considered in the development of healthy built environments. Indeed, meaningful stakeholder participation in land use decision making has been identified as one way that the built environment can promote human health and wellbeing through providing a sense of empowerment and inclusivity [1].

There are studies exploring different ways to incorporate the various agendas implicit in land use management and change. Stakeholders are frequently motivated by market imperatives, with cost benefit analyses that can demonstrate budget savings from healthy built environments in high demand as a way to engage different stakeholder groups. Publicity is also an important tool of engagement, implying that change must not just be quantifiably beneficial, but demonstrably so [80]. Finally, meaningful involvement of the community through consultation and education is often cited as key to bridging the gap between policy into behavioural change [62, 81, 82].

It is an easy task to argue that other stakeholders and agendas should be drawn into the healthy planning process. The reality of effectively actualising such broad collaboration is another matter entirely. It requires building and retrofitting firm foundations on which the health-built environment interdisciplinary relationship can rest and grow. The following section draws on literature that can assist in this endeavour. The research exposes some of the more controversial issues, including barriers to and opportunities for advancing and nurturing the healthy built environment interdisciplinary working relationship.

### 4.2. Working Together to Influence Policy Change

Two questions need to be answered before policy and practice changes can occur. The first relates to complicated interpretations of “evidence”: at what point do we consider that we have a strong enough case to challenge the policy status quo? The second question is perhaps less complex and relates to the way this evidence might be presented to the public and politicians to influence policy change. We now turn to consider these two questions.

#### 4.2.1. Evidence: “Are We Speaking the Same Language?” (See Page 49 in [83])

The question about evidence cuts to a core division between the health and urban planning traditions. Traditionally, the nature of evidence planners use to develop policy is different from that used by public health officials. Australian urban planning's early-to-mid 20th Century focus on greenbelt cities, for example, was based on a historical appreciation of the health benefits of open space for overcrowded and dirty cities [44]. Plans such as Sydney's County of Cumberland Plan and Perth's Endowment Lands project reflect this appreciation. Basing policy change on an “appreciation,” rather than hard evidence, would pose a problem for a public health-based intervention.

Establishing nonspuriousness by removing confounding variables (such as residential self-selection) and establishing time precedence through longitudinal research are regularly identified as the missing elements in evidence of the relationship between the built environment and health (see, e.g., Black and Macinko, [84]; Dunton et al. [85]). A lack of standardisation in measurement of environmental and health variables has also received attention as something that is missing in the research (see, e.g., Ball et al. [86]; Bodea et al. [87]). However, it must be recognised that the way people live and move around a place cannot be subject to the methods employed to produce the standard of evidence traditionally used to underpin health policy decisions. Recent discourse questions whether causal proof of the complex relationships between the built environment and health can ever be established. Increasingly, it is becoming obvious that more comprehensive ways to explore and understand the complex issues need to be embraced. This includes the use of case studies, in-depth observations, cost benefit analyses, environmental and social impact assessment, and demand analyses (e.g., see Ball et al. [88]; Coveney and O'Dwyer [89]; Thompson et al. [90]; Trayers et al. [83]).

Through embracing and exploring diverse methods, urban planning and health professionals must work to develop a mutually acceptable standard of evidence. There is research attempting to tackle this issue and bridge the gaps in understandings between the built environment and health for both policy makers and researchers. For example, Moodie uses Melbourne-based illustrations to develop a set of guidelines for ways public health researches can effectively communicate their research to policy makers [91]. He emphasises the need to seek out common interests and establish respectful relationships from the outset of the process. Bernard et al. study the impact any standardised notion of spatial scale might have on our ability to accurately examine the relationship between place and health [92]. They apply sociological theory to redefine neighbourhoods as domains through which people may have access to the resources required for healthy lifestyles. Cummins et al. discuss the mutually reinforcing relationship between people and place, calling for greater recognition of contextually sensitive policy [93]. Lawrence argues for integrative and interdisciplinary approaches to facilitate linkages between the built environment and health, with an acknowledgment of disciplinary expertise, as well as respecting expertise in other disciplines, as fundamental in creating shared understandings [94].

#### 4.2.2. Selling the Healthy Built Environment Concept

The second question, the way this evidence might be presented to the public and the politicians to influence policy change, is the focus of another emerging body of scholarship. 

Filion assesses barriers to the development of healthy built environments [95]. He concludes that when compared to other periods of significant urban change (such as the postindustrial shift to separate land uses or the post-World War II movement to low density), there is currently an insufficient critical mass of institutional and financial motivation to implement healthy built environments. Similar observations are made by Grant who concludes that the major obstacle to healthy built environment development in Canadian urban areas is weak political commitment combined with developer resistance [77]. Dodson et al. note the powerful role of market forces in preventing healthy eating policies in schools [64]. These studies demonstrate that the ability to communicate the evidence in ways likely to influence the intertwined forces of the market and politics will be key to effecting policy change. 

Cost benefit analyses of healthy built environment interventions are increasingly needed to satisfy the demand for economic justifications of policy change. There is an emerging body of research seeking to prove that the health and well-being benefits of healthy built environments (especially those resulting in reducing the health budget) outweigh the cost of their construction. Stokes et al., for example, simulated the potential yearly public health cost savings associated with investment in infrastructure for light rail (considered to be active transport). They were able to conclude a nine year cumulative public health cost savings of US$12.6 million [96]. 

In addition, a body of research is developing which analyses market demand for and developer perspectives of healthy built environments. Carnoske et al., for example, surveyed 4,950 real estate agents and 162 developers in the USA [97]. Their aim was to assess factors influencing homebuyers' decisions, as well as incentives and barriers to developing healthy built environments. The research concludes that there is a perception of increased residential demand for healthy built environments. However, developers, in particular, perceive significant barriers to creating these communities [97]. The limitations of local government politics and regulations perceived by developers were also confirmed by other literature (see, e.g., Levine and Inam [98] and Bjelland et al. [99]). In a larger-scale study of actual consumers, Handy et al. analysed data from two surveys from 2003 (*n* = 5,873) and 2005 (*n* = 12,630) to assess changes in consumer support for “Traditional Neighbourhood Design” (TND) [100]. Surveys described a traditionally designed neighbourhood and asked respondents “how much would you support the development of communities like this in your area?” The study concludes that support for TNDs had increased from 44 to 59 percent from 2003 to 2005. In a review of over 50 relevant studies, Shoup and Ewing examine the economic value of outdoor recreation facilities, open spaces, and walkable community design [15]. Their synthesis of the research concludes that open spaces such as parks and recreation areas can have a positive effect on residential property values and justify higher property tax revenues for local governments. The research also concludes that compact, walkable developments can provide economic benefits to real estate developers through higher home sale prices, enhanced marketability, and faster sales or leases than conventional development. Interesting market demand research by the Australian Heart Foundation (2011) reveals consumer preference for healthy built environments [101]. This national telephone survey (*n* = 1,400) found that people valued environments where they could walk to local shops and services, use public transport, and access open space for recreation. It was reported that “these features were rated more highly than having a two car garage and large backyard- features more typically associated with car oriented suburban neighbourhoods (or urban sprawl)” [101]. Such findings may well influence developer provision of healthy built environments to meet consumer preferences.

## 5. Conclusion

In summary, research on the link between human health and the built environment justifies increased theoretical and professional recognition of health as a primary motivator for urban planning. The foundations for this have already been laid by existing synchronicity between health and urban planning theories. 

Beyond theory, a reinvigorated health focus for urban planning can further legitimise the principles and practices planners have long recognised as good practice. Health is a driver that can take the urban planning agenda forward. Accordingly, the relationship between health and urban planning professionals needs to be nurtured from both theoretical and practical perspectives. Case study examples of successful collaborations can be found on various websites. In Australia there are the websites of the New South Wales Premier's Council for Active Living, Victoria's VicHealth, the National Heart Foundation and Healthy Places and Spaces. In the USA, there is the Active Living Research Program by the Robert Wood Johnson Foundation, the Centres for Disease Control and Prevention and the U.S. National Physical Activity Plan. This literature provides a rich and grounded understanding of opportunities for implementing healthy built environments, showing how common barriers are being addressed and overcome, as well as inspiring new collaborations.

While it is true that health and urban planning were successful partners a long time ago, this was not within the contemporary neoliberal framework of academic, political, and policy silos. An effective healthy built environment profession today rests on building a respectful relationship out of mutual understanding and fruitful, practical engagement across these silos. Scholarship on how this is happening is emerging, and this body of research should act as a forum for the interdisciplinary exchange of examples, ideas, and commentary. These innovative lines of communication must be supported and catalogued for ongoing reference. We believe there will always be a need for professionals working in this area to take stock of their achievements and communicate what has worked and what has failed.

This discipline area is in its infancy. It is our hope that it is a discipline that develops to create built environments that can better promote human health and well-being. 
